# Biomimetic Anisotropy for Directional Transport of Liquid and Solid Samples

**DOI:** 10.3390/biomimetics11030181

**Published:** 2026-03-03

**Authors:** Adem Ozcelik

**Affiliations:** Department of Mechanical Engineering, Aydin Adnan Menderes University, Aydin 09100, Türkiye; aozcelik@adu.edu.tr

**Keywords:** biomimetic anisotropy, rectified transport, directional wetting, capillary-pressure bias, elastocapillary coupling

## Abstract

Biomimetic anisotropy is defined as intentionally engineered, nature-inspired directional differences in structure, chemistry, roughness, stiffness, or pore architecture. These directional differences lower transport resistance in one direction relative to the opposite direction, which results in rectified transport. In this review, anisotropy design is synthesized across surfaces, porous materials, and soft systems, with transport considered for droplets, low-surface-tension liquids, particles, and soft objects. Biological inspirations are summarized first, and the design lessons that can be transferred to engineered platforms are then extracted. Key anisotropic architectures are classified next, including ratchets and sawtooth textures, bristle- or setae-like fibrillar arrays, grooves and wedges, asymmetric pores and membranes, chemically patterned surfaces, and hierarchical micro–nano combinations. Practical fabrication methods and material choices are reviewed thereafter, spanning micro- and nanofabrication, additive manufacturing, coatings and surface modification, and responsive soft matter. The field is then organized mechanistically around how anisotropy generates directionality through contact-line pinning asymmetry, curvature-driven capillary pressure bias, compliance and elastocapillary coupling, and active rectification under oscillatory forcing. Finally, these mechanisms are connected to application needs in pump-free microfluidics and sampling, long-distance open transport, environmental water management, and fouling-prone self-cleaning systems. Throughout the review, design-to-function links are emphasized, and open challenges are highlighted, including durability under real fluids and contaminants as well as scalable manufacturing and integration.

## 1. Introduction

Moving liquids, particles, and even soft objects in a controlled direction is a basic need in many technologies. In microfluidics and lab-on-a-chip systems, it can reduce or remove the need for external pumps [1,2]. In materials used in daily life like textiles, membranes, and coatings, the ability to directionally move droplets and liquids can help with water collection, moisture management, separation, and anti-fouling. Nature already does this very well. Many natural surfaces and structures are not the same in every direction. They have built-in directionality, so water or other matter tends to move one way more easily than the other [3]. This type of directionality has motivated a large body of biomimetic work, where researchers copy (or adapt) natural design rules to make artificial surfaces and materials with one-way or preferred-direction transport [4,5].

In this review, the term biomimetic anisotropy is used to mean nature-inspired directional differences that are intentionally built into a surface or material so that transport is easier in one direction than another. Anisotropy means that the structure, chemistry, roughness, stiffness, or pore architecture is direction-dependent and that direction dependence creates rectified net transport. In many cases, the goal is not to apply more energy but to use geometry and interfacial physics so that the system prefers a direction. The recent literature describes this idea as passive, non-energy-consuming directional liquid transport, where driving forces come from asymmetric chemical, roughness, and curvature gradients [6]. This framing is useful because it ties bioinspired designs to clear physical sources of driving force.

Most early and widely studied examples focus on droplets and wetting. Directional wetting and droplet motion can be achieved by using one-dimensional (1D) or directional micro/nanostructures, which create different spreading or pinning behavior along different axes of the surface [5]. Building on this base, later work expanded into superwettability design (superhydrophobic/superhydrophilic, superoleophobic/superoleophilic, and related combinations), where extreme wetting states can amplify directionality and improve droplet control [6,7]. These surface-based approaches are now connected to broader applications, including droplet routing, condensation control, self-cleaning, and liquid handling for assays.

A second major branch moves beyond flat surfaces into thin porous materials (fabrics, electrospun membranes, and meshes). Here, anisotropy is often built through the thickness, for example by combining layers with different wettability or pore structure. This enables directional transport through a membrane, not only along a surface. This is attractive because directional liquid motion driven by the substrate is valuable for microfluidic handling and water collection, and recent work has demonstrated directional transport across thin porous materials with expanding application opportunities [8]. Importantly, porous materials introduce additional design control like pore connectivity, layer stacking, and breakthrough pressure differences, which can create strong one-way behavior even against gravity in some designs.

A third branch extends biomimetic anisotropy to transport beyond small droplets toward macroscale objects and soft matter in liquids. This is much harder, especially underwater, because drag and low-Reynolds-number constraints limit what passive surface texture alone can do. Wang et al. point out that while many artificial anisotropic interfaces can move droplets, transporting a macroscale object underwater remains challenging [3]. They show that anisotropic microcilia arrays with an asymmetric stroke can generate directional flow and move a centimeter-scale hydrogel slice underwater. This example is important for the field because it demonstrates that anisotropy can be coupled with periodic actuation (here, magnetic) to create strong directional transport at a larger scale while still keeping the direction-setting role of the anisotropic structure.

Finally, anisotropy-based transport is not limited to visible droplets and objects. Similar direction matters ideas also appear at the nanoscale in biology. For example, structural anisotropy in biological systems can bias transport processes, such as protein movement across nuclear pores, where the local structure and mechanics of cargos influence transport rates and directionality [9]. While the physics and environment differ from surface wetting, this reinforces a central message that anisotropy is a general strategy for controlling transport across scales and across different kinds of matter including liquids, soft solids, particles, and biomolecules.

**Figure 1:**
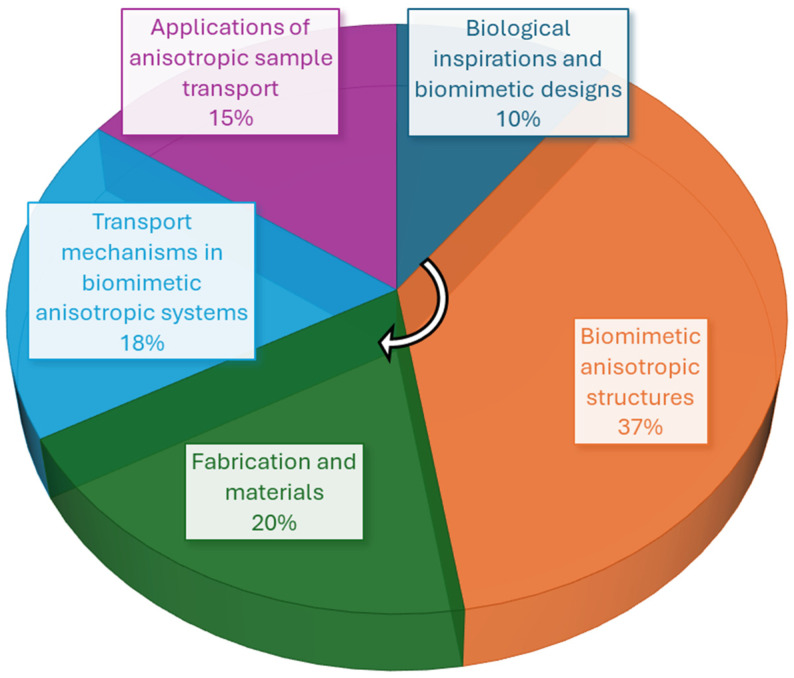
Literature distribution across the main review themes, quantified by the number of unique cited references in each section.

Based on the aforementioned literature, this review focuses on how biomimetic anisotropy is designed and how it produces directional transport. We emphasize types of anisotropy that are built into the material, transport mechanisms, and applications from droplet routing and moisture management to separation, underwater transport, and bio-interfaces [6,8]. Finally, we provide an outlook on the remaining challenges and future opportunities for the field. To help readers navigate the review and see where research activity has concentrated, Figure 1 provides a quantitative snapshot of the literature distribution across the major themes covered in this article. The chart summarizes the relative emphasis of the cited work across biological inspirations, anisotropic architectures, fabrication and materials, mechanistic principles of rectification, and application areas, offering a simple map that mirrors the review structure.

## 2. Biological Inspirations and Biomimetic Designs

### 2.1. Natural Systems That Bias Transport

**Figure 2:**
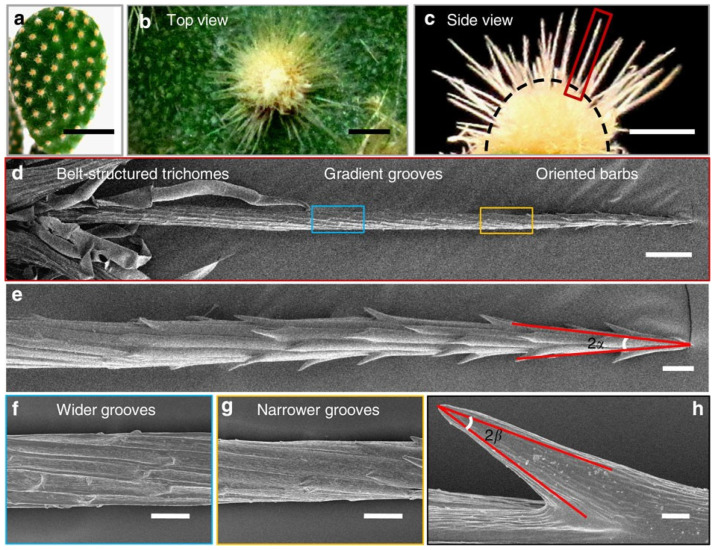
Hierarchical spine–trichome architecture of *Opuntia microdasys*. Optical imaging (**a**–**c**) shows the stem surface populated by evenly spaced spine–trichome clusters (**a**), with individual clusters resolved in top (**b**) and side (**c**) views where spines are seen emerging from trichome bundles. SEM analysis (**d**–**h**) reveals a representative spine organized into distinct zones (**d**): a tapered tip bearing aligned barbs (**e**) characterized by an apex angle (2α), a grooved midsection with a clear gradient in surface texture (**f**,**g**), and a basal region associated with belt-like trichome structures. Higher-magnification views indicate that microgrooves are broader and more widely spaced near the base (**f**) and become narrower and denser toward the tip (g). A single barb at the tip is shown at high magnification in (**h**), where the barb apex angle (2β) is indicated. Scale bars: 5 cm (**a**), 500 μm (**b**,**c**), 100 μm (**d**), 20 μm (**e**–**g**), and 2 μm (**h**). Reproduced from [12] under the Creative Commons Attribution—Non-Commercial—ShareAlike 3.0 Unported License (CC BY-NC-SA 3.0).

Many living systems move liquids, particles, or cells in preferred directions by using anisotropy in structure, chemistry, or motion. A common example is directional wetting and drainage on surfaces that have aligned micro–nano features. Plants and insects often combine multiscale roughness with surface chemistry to control where water collects and how it moves, so transport is not the same in all directions [4]. Another well-known example of wetting is spider silk, where repeated structures along the fiber create surface energy and curvature differences that drive condensation and droplet motion toward specific locations [10]. Other natural ideas often used in biomimetic design include cactus spines (Figure 2) and pitcher plant peristomes, where shape and texture guide water to move directionally and continuously [11,12].

Animals also show frictional anisotropy, where motion is easier in one direction than another. Gecko and insect attachment systems use oriented setae and hierarchical contacts so that adhesion and friction depend strongly on the direction of loading and peeling [4]. This kind of directional grip is a direct biological lesson for surfaces that must allow one-way motion, controlled release, or asymmetric drag.

A third class of inspirations is based on fields of moving or flexible elements such as cilia-like structures. In biology, cilia are organized arrays that generate directional flow and transport by coordinated beating; their structure and organization are closely tied to function [13]. Biomimetic versions of cilia-like arrays can create net transport in liquids and can be used to move objects or fluids when driven by an external stimulus [14]. Finally, transport bias also exists at smaller biological scales. Inside cells, directional cargo transport comes from anisotropic tracks and motor activity, and the rules of that transport (direction choices, switching, and regulation) are well studied [15]. These cellular examples are useful because they show that directionality can come from geometry and from controlled, energy-driven processes.

### 2.2. From Inspiration to Engineering Translation

**Figure 3:**
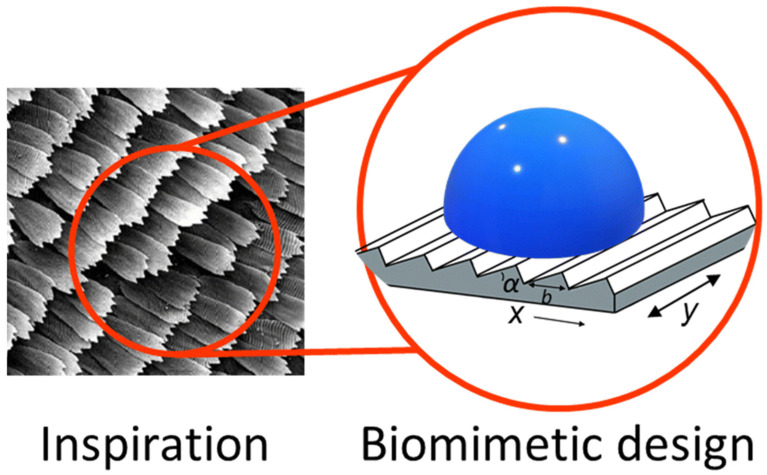
An example of inspiration to engineered design. Butterfly wing scales (left) are shown as the biological reference that motivated the selected surface geometry. The engineered unit cell is defined by the blaze angle (α) and blaze length (b), which are the key geometric parameters. Coordinate axes are indicated, with x assigned as the primary transport direction. The y-axis is shown without a positive direction because the surface is symmetric along y. Reused from [18] under the Creative Commons Attribution—Non-Commercial 3.0 Unported Licence (CC BY-NC 3.0).

When engineers translate biological anisotropy into devices, they usually copy a small set of transferable features like geometry, hierarchy, compliance, and surface chemistry (Figure 3). Studies of anisotropic wetting surfaces highlight how one-dimensional textures and directional structures can create different advancing/receding behavior depending on direction, which is a core mechanism behind biased droplet motion [16]. Related studies on superwettability-driven droplet transport describe how designers combine texture, chemistry, and sometimes external triggers to produce repeatable directional motion [17]. Practical implementations also extend beyond rigid surfaces. Directional fluid transport in thin porous materials shows how anisotropy can be built into fibrous or porous networks to drive one-way wicking and pumping-like behavior in flexible formats [8].

Robustness and integration usually have to be engineered. Natural surfaces are self-renewing and operate in specific environments, while engineered systems must survive abrasion, fouling, and manufacturing variation. Work on bioinspired directional surfaces emphasizes that translation often depends on scalable fabrication routes and on modeling tools that link microstructure to function. Broad reviews also show that the field is moving toward designs that work under more realistic conditions (such as underwater, dirty environments, or textiles) and toward topological approaches that keep directionality even when liquids fully wet the surface [19].

Another important translation path is using anisotropy inside soft materials. Biological materials often rely on oriented structures to achieve direction-dependent properties, and this idea is a general design rule across many biological systems [20]. In engineered hydrogels, anisotropic structure can be used to produce controlled deformation and actuation, which then creates transport bias [21]. Similar ideas appear in biofabrication, where aligned features and controlled microenvironments are used to guide cells and tissues, which is essentially transport bias applied to living matter [22]. Finally, some translations follow the cell biology more directly such that directional transport can be built from energy-driven processes and molecular design, as shown by work on autonomous chemically fueled molecular motors [23].

## 3. Biomimetic Anisotropic Structures

### 3.1. Ratchets and Sawtooth Topographies

**Figure 4:**
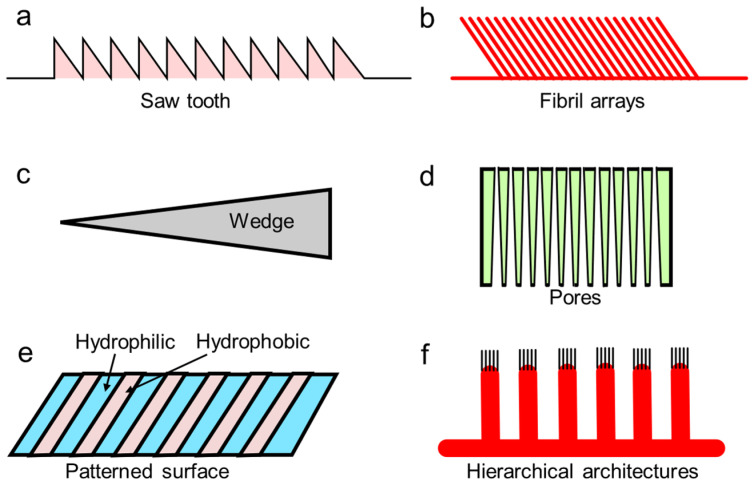
Schematic depiction of various anisotropic structures. (**a**) A saw tooth/ratchet structure. (**b**) Fibril array/setae-like structures. (**c**) A wedge geometric structure. (**d**) Cross-section of a material with anisotropic pores. (**e**) A patterned surface with hydrophilic and hydrophilic stripes. (**f**) An example of a hierarchical structure with two level roughness.

Ratchets and sawtooth topographies are surface patterns that have a left–right asymmetry (Figure 4a). The idea is that when a droplet, film, particle, or even a soft body meets an asymmetric texture, it can face different resistance depending on which way it tries to move [2,16]. In biomimetic transport, these patterns are used as a physical way to bias motion without adding valves or complicated moving parts. Asymmetric microstructures including ratchet-like geometries are a core strategy for directional liquid transport, either working on their own or combined with external stimuli such as heat, light, or electric fields [24].

**Figure 5:**
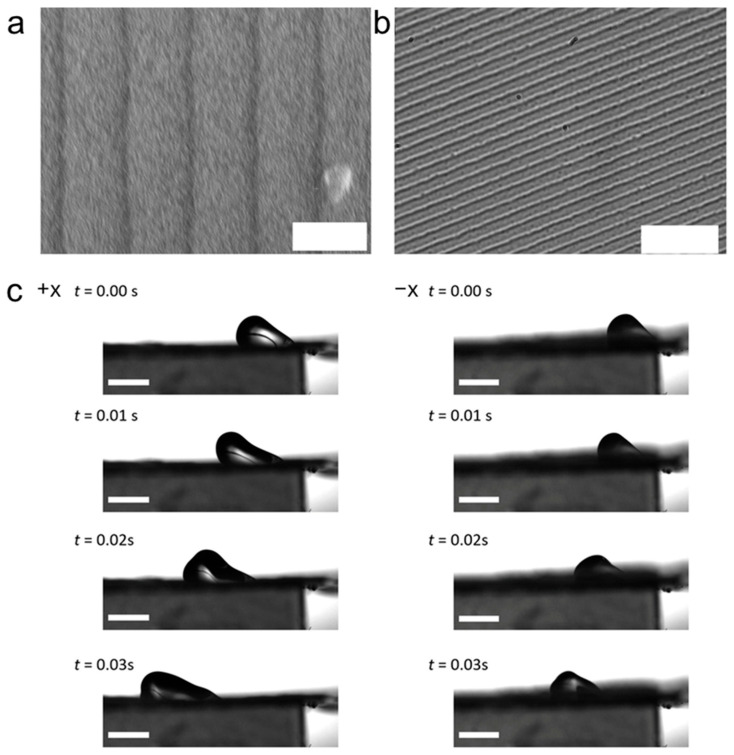
Surface replication and anisotropic droplet transport on imprinted patterns. (**a**) High-magnification SEM of a PDMS replica surface (15,000×; scale bar: 2 μm). (**b**) Bright-field optical micrograph of an imprint produced in a Triton X-100-modified acrylate resin (100× objective; scale bar: 10 μm). (**c**) Time-sequenced still images of a droplet traveling across the Triton-modified acrylate lithograph, showing direction-dependent mobility; motion toward the −x direction is slower than motion toward +x (scale bars: 2 mm). Reused from [18] under the Creative Commons Attribution—Non-Commercial 3.0 Unported Licence (CC BY-NC 3.0).

A key reason ratchets work is contact-line friction and pinning. The contact line where solid, liquid, and air meet does not slide smoothly on a real surface. Instead, it sticks and slips, and the barrier for depinning can depend on direction when the topography is asymmetric. A clear example is the study on slanted sawtooth surfaces, which reports asymmetric contact-line friction caused by the sawtooth geometry [18]. This kind of directional friction is the microscopic basis for macroscopic one-way droplet response (Figure 5). The droplet can more easily advance in one direction than the other under the same forcing, because the contact line meets different slopes/sharp edges depending on direction.

Another closely related mechanism is directional capillary holding and guided motion inside asymmetric grooves. This design can be framed as a ratchet-like surface with oriented open-wedges, aimed at stronger capillary retention and controlled droplet manipulation [25]. Open-wedge geometries matter because they set local curvature and capillary pressure in a direction-dependent way. The meniscus shape and the way the contact line anchors at wedge edges can differ depending on the approach direction. In practice, this means that the same drop can be easy to move one way while resisting motion the other way, or it can be trapped and released in a controlled direction by small changes in forcing.

Design-wise, ratchets and sawtooth patterns are not fixed for all applications. Performance depends on geometric ratios such as height, pitch, tooth angle, sharpness. In addition, the wetting state is also important (for example, whether the liquid penetrates texture or sits on top of trapped air is critical for the transport of droplets). Ratchet-like and asymmetric microstructures can be discussed alongside other directional designs like gradients and Janus wettability because real systems often combine multiple sources of anisotropy to achieve reliable transport over a wider range of droplet sizes, speeds, and contamination conditions [24]. In other words, the ratchet geometry is usually one part of an anisotropic system. Surface chemistry, hierarchy, and robustness choices also play important roles in actual applications.

Finally, it is useful to view ratchets as a general transport concept, not only a droplet concept. The same asymmetric topology idea appears in systems that bias the motion of soft matter or living matter. For example, a phase ratchet framework can flip velocities of living cells and inert beads [26], showing that ratchet principles can select or bias motion even when the moving matter is not a droplet. This is important because it supports a broader claim that anisotropic, ratchet-like structures are a cross-cutting tool for directional transport across droplets, particles, and biological materials, as long as the interface supplies a direction-dependent barrier or driving force.

### 3.2. Bristles, Setae, and Directional Fibrillar Arrays

Bristles, setae, and fibrillar arrays are one of the most direct ways to build anisotropy into a surface (Figure 4b). The main idea is that a surface covered with tilted or oriented fibers does not feel the same when you move across it in opposite directions. In biological systems, this is used to control grip, release, and friction. In engineered systems, the same geometry and compliance can be used to bias the motion of solids through anisotropic friction/adhesion and to bias the motion of liquids through direction-dependent pinning and guided spreading [27,28].

**Figure 6:**
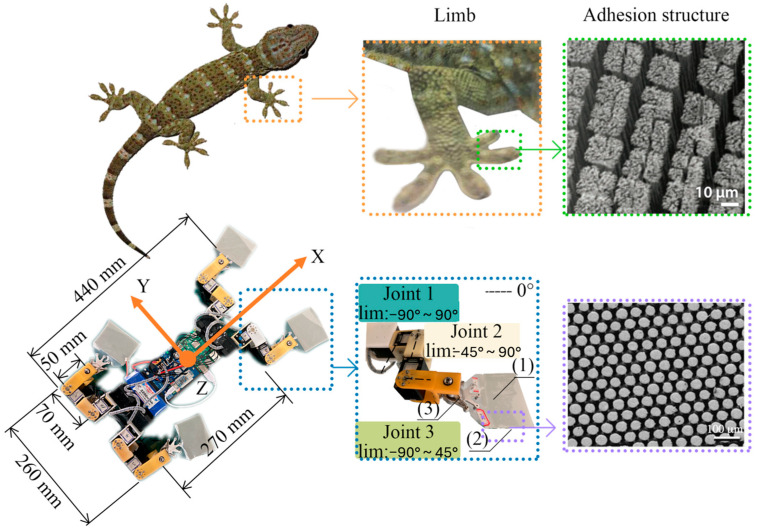
Gecko-inspired wall-climbing robot layout and dry-adhesion foot module. The overall body and limb configuration is shown together with joint workspaces and the neutral (0°) reference positions used for kinematic definition. The adhesion concept is illustrated by comparing gecko setae with the robot’s dry-adhesion pad, where the contact material is polyvinyl siloxane (PVS). The foot assembly is composed of a polyvinyl chloride (PVC) backing layer, a PVS adhesive layer, and a passive compliance/realignment element implemented with a ball bearing, and a return spring. Reused from [30] licensed under the Creative Commons Attribution—4.0 International License (CC BY 4.0).

For solid transport, directionality often comes from how fibers bend and how contact area changes with loading direction. Gecko-inspired designs aim to reproduce controllable attachment by using aligned microstructures that engage strongly in one direction but peel more easily in the reverse direction [27]. A practical example is provided by the IBSS–8 wall-climbing robot, where a gecko-like PVS dry-adhesion pad is paired with a control strategy that drives detachment by increasing the peeling angle toward π, thereby minimizing abrupt changes in normal adhesion force during release (Figure 6). This shows that directional attachment can be strengthened by predictable peeling dynamics, which supports stable locomotion even when the adhesion state varies across steps or surfaces. This is not only a strong vs. weak adhesion situation. It is also a stability matter such that if the contact elements engage in a predictable way, sliding becomes more stable and can avoid sudden stick–slip.

A direct example is setal-array sliding on rough surfaces, where friction reduction and motion stability are treated as outcomes of how an array of setae interacts with surface roughness during sliding [29]. This supports a practical design idea for fibrillar arrays: the goal is not simply to maximize friction or adhesion but to tune the array so it maintains stable contact over realistic roughness and loading paths.

The same anisotropic fiber logic is useful for wetting and droplet transport because the moving contact line meets different barriers depending on direction. Tilted pillar arrays are a clean engineered analogue of tilted fibers such that the pillar tilt gives a preferred direction for droplet spreading and transport, because the contact line can advance more easily along the with-tilt direction than against it [31]. This type of design is especially relevant when directional motion without closed microchannels, because the droplet can be guided directly by the surface texture. Hierarchical leaf-like textures add another layer to this. When micro/nanostructure controls wettability state transitions, the surface can switch between regimes that favor pinning versus spreading, which changes how easily directional transport can be triggered or stopped [32]. Put simply, fibrillar and pillar arrays set the geometric direction. The wetting state determines whether that directionality becomes a strong one-way effect or a weaker bias.

When fibrillar arrays are made active, they move from direction-biased surfaces to direction-generating interfaces. Artificial cilia systems are built to produce net flow and transport by using non-reciprocal beating, and many designs focus on how to program coordination so the array creates useful pumping, mixing, or transport rather than local stirring only [33]. Directional liquid manipulation can also be framed as a function of structure across dimensions meaning that geometry and arrangement of cilia-like elements set where liquid can go and how fast it drains, even before one chooses the actuation method [34]. At the biological level, coordinated beating is not optional. Epithelial cilia need coupling to sustain directional beating across a tissue, which is a strong reminder that array-level interactions matter as much as single-element shape [35]. These same principles apply in engineered cilia arrays that achieve programmed nonreciprocal motion and metachronal coordination to enhance transport performance at low Reynolds numbers [36].

In proton exchange membrane fuel cells, water management depends on fast, directional drainage, and cilia-inspired channel concepts are used to speed up and control the direction of water removal [37]. This application is useful for a broader perspective because it shows what engineering translation looks like for fibrillar anisotropy. The design is judged by drainage speed, reliability under operating conditions, and integration into a functional system, not only by droplet motion on a test surface. Across passive and active versions, the main design levers repeat such that fiber/pillar tilt and spacing set the directional bias. Compliance sets stability, and coordination (for active arrays) determines whether the structure produces local motion or a strong net transport direction [29,31,35].

### 3.3. Grooves, Wedges, and Capillary Diodes

**Figure 7:**
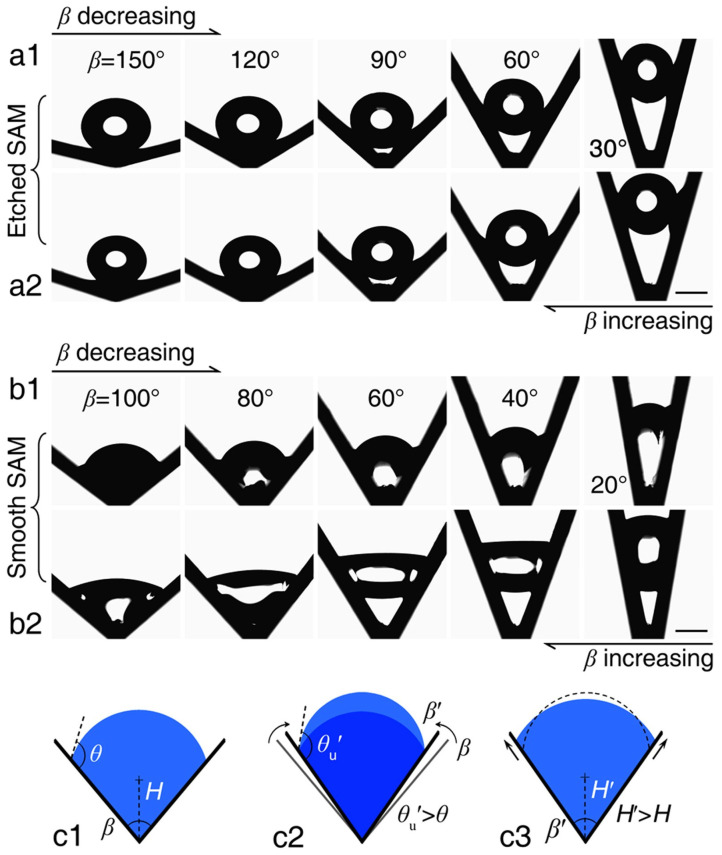
Resting-mode switching in V-grooves and the role of contact-angle hysteresis. Droplets in V-shaped grooves exhibit two stable configurations—immersed (IM) and suspended (SU)—and the switching between these states depends on the groove half-angle β and surface hysteresis. For an etched SAM groove, a switch from IM to SU occurs during *decreasing* β at about $\beta \approx 106^{\circ }$, while the reverse switch from SU to IM appears during *increasing* β near $\beta \approx 109^{\circ }$ (**a1**,**a2**). For a smooth SAM groove, the IM to SU transition is not observed during decreasing β, whereas SU to IM occurs during increasing β at approximately $\beta ∼70^{\circ }$ (**b1**,**b2**), highlighting a strong hysteresis effect. Panels (**c1**–**c3**) illustrate the proposed mechanism: slight narrowing of the groove deforms the upper meniscus, increases the local contact angle toward the advancing angle, and triggers upward motion of the upper contact line, which initiates the IM-to-SU transition. Scale bar: 1 mm. Reused from [39] licensed under the Creative Commons Attribution—4.0 International License (CC BY 4.0).

Grooves and wedges provide directionality by shaping the capillary pressure along a path (Figure 4c). When a wetting liquid sits in a corner, the meniscus curvature can stay high and keep pulling the liquid forward, even without any external pumping. This is the basic corner effect idea. The key controls are the wedge (or groove) angle, the intrinsic contact angle, and how the cross-section changes along the track. If the geometry supports stable wetting in the corner, liquid can self-propagate; if not, the flow breaks or stops [38]. A related example is provided by droplet transport in a simple V-shaped groove, where the motion direction is governed by the groove cross-sectional angle and the inner-wall wettability [39]. Two resting modes were reported, with immersed droplets at larger groove angles and suspended droplets when the geometry satisfies β < 2θ − π (Figure 7). Importantly, contact-angle hysteresis was shown to shift or suppress the expected mode transition, which changes whether the droplet remains pinned near the groove bottom or lifts into the suspended state during transport.

A capillary diode adds a second element: it keeps the forward driving force but blocks reverse motion by pinning the contact line at a geometric step or junction. A practical way to do this is to connect wedge-shaped units in series so the liquid always sees a gradual expansion in the forward direction but an abrupt angle/width change in the reverse direction. The reverse junction increases pinning, so the meniscus does not easily move backward, while the forward direction still benefits from a sustained Laplace pressure bias. A clear example is a 3D wedge-shaped diode made with laser fabrication, where connected wedge units generate a pressure gradient and intentional pinning, enabling fast and long-range unidirectional transport across different wetting conditions and viscosities [40]. This diode framing also matches how recent reviews define liquid diodes as repeated wedge/sawtooth capillary elements that generate sustained forward motion and reverse pinning over long distances [41].

Beyond the diode concept, grooves and wedges are useful because they can be integrated into larger functional devices. For example, unidirectional capillary transport can be combined with porous and hierarchical structures to create strong one-way liquid delivery for thermal systems. One recent device-level demonstration uses V-grooves plus asymmetric wedge-like microcavities inside a microporous hierarchical structure to enforce unidirectional wicking. When paired with phase change, this enables strong heat-flow rectification over long distances [42]. This shows a broader point that grooves/wedges are not only about moving droplets on open surfaces but also about controlling circulation and supply in engineering systems where backflow is not desired.

Groove/wedge ideas also translate well to lubrication and low-friction transport, where one-way spreading can stabilize a lubricating film in the desired direction. A wedge-shaped lyophilic pattern placed on a strongly repellent background is one way to force liquid to stay on the intended path and resist undesired lateral spreading or reverse drift. In tribology contexts, this is discussed as a strategy to manage liquid distribution and motion direction using a patterned combination of affinity and repellency [43].

In parallel, the corner-effect perspective is useful for making sense of when grooves/wedges will keep pulling liquid forward and when they will fail, especially when the track includes repeated corners, turns, or junctions that can either help (extra capillary pull) or prevent (excessive pinning) depending on the geometry and wetting regime [44].

Finally, recent papers emphasize that groove/wedge diodes are attractive because they can be made in scalable ways (laser cutting, molding, printing) and can be tuned by geometry rather than relying only on delicate surface chemistry. At the same time, there are also some engineering tradeoffs. Strong reverse pinning helps rectification, but too much pinning can trap the front. Higher forward speeds often require larger channels, but that can weaken the capillary pressure. In addition, performance can drop when contamination, damage, or long-term wetting changes occur. These issues show up repeatedly in anisotropic wetting and unidirectional transport, including groove- and wedge-based tracks and diode layouts [38,41,45].

### 3.4. Asymmetric Pores and Membranes

Asymmetric pores and membranes create directionality by making it easier for liquid to enter from one side than the other (Figure 4d). The most common approach is a Janus design, where the two sides have different wettability (for example, hydrophobic on one side and hydrophilic on the other) while pores connect the two sides. In this setup, the same pore network enable one-way transport because the capillary entry condition is different depending on which side the droplet starts from. In the well-known Janus membrane example by Tian and colleagues, the membrane shows droplet and flow gating that depends on direction, which is useful when passive one-way control without valves or external power is desired [46].

A key point made across the aforementioned literature is that directionality is not only about chemistry on two faces. It also depends on pore geometry, pore size distribution, and how the interface pins inside the pore network. Studies on Janus membranes emphasize that practical liquid diode behavior usually comes from coupling surface contrast with a pore architecture that makes capillary barriers strongly different in the two directions, so the forward direction has a lower entry pressure than the reverse direction [47,48,49]. This idea explains why many high-performance designs combine asymmetric wettability with micro/nano-structuring, hierarchical roughness, or conical/needle-like pores rather than relying on a flat coating alone.

Electrospun fibrous membranes are a major engineering approach because they give high porosity and a tunable pore structure while also allowing one-sided functionalization. For example, electrospun nanofibrous membranes are adapted for oil/water separation by building hydrophilic/hydrophobic asymmetry, often targeting both directional penetration and fouling resistance under realistic mixtures and emulsions [50]. In these systems, directional transport is often framed as a way to achieve gravity-driven separation or self-driven penetration in one direction while blocking backflow, which is useful for separation systems that should run without pumps.

At smaller length scales, asymmetric pores become nanofluidic channels, where surface charge and confinement dominate transport [51,52]. For example, a channel’s internal surface state can be engineered to bias transport and enable multiple functions in a single platform [53]. More broadly, the rectification can come from asymmetric charge, asymmetric geometry, or spatially patterned functional groups, producing diode-like ionic behavior that is conceptually similar to one-way wetting but governed by electrostatic interactions rather than macroscopic capillarity [54]. Recently, it was shown that biomimetic nanofluidic channels can be designed for selective ion transport and separation applications, illustrating that asymmetry can be implemented as a structured transport pathway with strong selectivity, not only as a two-faced coating [55].

### 3.5. Chemically Anisotropic and Wettability-Patterned Surfaces

Chemically anisotropic surfaces, as schematically depicted in Figure 4e, create directionality by making one region easier to wet than another [56]. This can be achieved by changing surface chemistry while keeping the surface shape the same or by combining chemistry with texture. The basic idea is that a droplet on a patterned surface sees different advancing and receding contact angles at its front and back, so the force balance is biased in one direction. Many studies treat these surfaces as passive actuators for droplets. They have no moving parts, but a built-in asymmetry that can guide motion when a droplet is added, tilted, vibrated, condensed, or merged with another droplet [57].

A common design patterns hydrophilic tracks in a more hydrophobic background or creates step changes in wettability that define where a liquid can spread and where it stops. These patterns can steer droplets, split them, or define preferred flow paths. For microfluidics, one clear use is controlling where water moves in a channel or on an open surface, using chemical patterns that impose an easy direction and a hard direction for wetting and motion [58]. Patterning is also used as a practical manufacturing tool. For example, maskless laser-based functionalization on polymer coatings has been demonstrated for large-area wetting/dewetting patterning, with direct relevance to flow patterning and cell patterning [59]. These fabrication-focused studies matter because chemical patterns often fail in practice if they cannot be produced reliably over large areas or if the patterns drift over time due to contamination or aging.

Beyond fixed tracks, another important class is wettability gradients and ratchet-like chemical landscapes, where the surface energy changes continuously or in repeated asymmetric units [60]. These surfaces can produce self-propulsion behaviors under specific driving conditions, for example, when droplets coalesce or when the surface is refreshed by condensation. A clear example is the use of robust wettability gradient surfaces designed to support ratchet-like droplet motion, where the gradient helps the droplet overcome pinning in one direction more easily than the other [61]. Gradients are also used in thermal settings. In dropwise condensation, local wetting contrast and gradients can change nucleation, droplet growth, and droplet removal, which directly affects heat transfer. Wettability gradients can be framed as a way to actively bias condensation outcomes, while real performance depends on hysteresis, defect sensitivity, and long-term stability [62].

Chemical anisotropy can also be created dynamically, not only by fixed surface patterning. One example is surfactant adsorption on hydrophobic surfaces, where local changes in surface condition can enable droplet motion and even allow droplets to be guided or manipulated in ways that resemble pickup and release behaviors [63]. Related work describes droplet manipulation on hydrophobic substrates by surfactant-mediated effects, showing that chemical state and not only geometry can be used to control droplet behaviour [64].

### 3.6. Hierarchical Multiscale Architectures

Hierarchical (multiscale) architectures (Figure 4f) are used when a single length scale cannot provide stable directionality across real conditions. In transport problems, the small features mainly set the local wetting state and pinning behavior, while the larger features set the preferred pathway and the overall driving asymmetry. Multi-scale structures can shift the wetting-state thresholds (for example, when Cassie–Baxter versus Wenzel states are favored), which changes whether a droplet can move, stop, or switch modes as it meets new features along a path [65]. This is why hierarchical designs often perform better than single-scale textures when droplets change size, speed, or composition.

**Figure 8:**
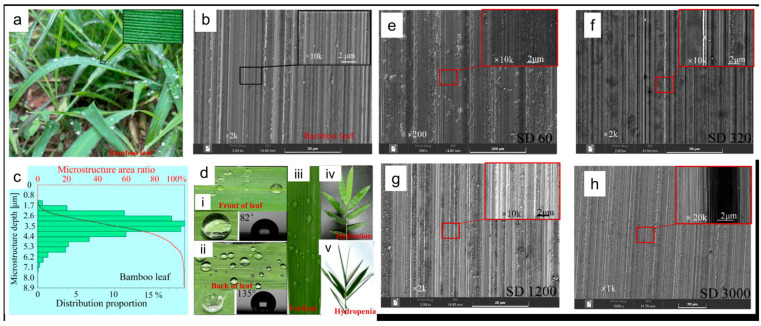
Natural bamboo leaf hierarchy and microground silicon analogs. (**a**) Photograph of a bamboo leaf showing the vein-aligned texture. (**b**) Environmental SEM highlighting the ordered groove field on the fresh leaf surface. (**c**) Abbott–Firestone (bearing area) curve summarizing the height/depth distribution of the leaf topography. (**d**) Representative images illustrating the hydration-dependent shape change of the leaf. (i, ii) photographs of a bamboo leaf under hydration and hydropenia conditions, respectively; (iii) droplets pinned on a vertically oriented leaf; (iv, v) wetting behavior on the front (adaxial) and back (abaxial) sides of the leaf, respectively. (**e**–**h**) SEM views of bamboo-leaf-like hierarchical-structured (BLHS) Si surfaces produced by one-step microgrinding using diamond wheels with different grain sizes: SD60 (**e**), SD320 (**f**), SD1200 (**g**), and SD3000 (**h**). Reproduced from [32] under the Creative Commons Attribution—4.0 International License (CC BY 4.0).

An illustrative case is the bamboo-leaf-like hierarchical-structured (BLHS) silicon surface formed by one-step microgrinding, where micro–nano grooves produced a stable transitional hydrophobic state without additional surface chemistry (Figure 8) [32]. Contact angles increased to about 97° and remained essentially stable after droplet impacts, indicating that the multiscale texture can preserve a functional wetting state under dynamic loading. The wetting transition was attributed to the hierarchical topography rather than chemical modification, supporting the idea that multiscale architecture can shift practical wetting thresholds.

Many hierarchical designs start from specific wettability surfaces, where micro/nanostructure and surface chemistry work together to create robust wetting states. For example, lotus-leaf-like superhydrophobicity, fish-scale-like underwater superoleophobicity, and pitcher-plant-like infused surfaces are built from cooperating structural levels to guide droplets in preferred directions when combined with asymmetric layouts [66]. In this view, directionality is rarely produced by just one feature. It is more often produced by a system that includes a stable local state (low adhesion or controlled pinning), a repeated anisotropic element (tilt, wedge, or ratchet-like unit), and a larger-scale route that prevents backflow or spreading into undesired regions.

Several studies use hierarchical architectures to improve durability and function under harsh environments, which is important for transport surfaces that must work beyond a clean laboratory setting. A lotus-inspired multiscale superhydrophobic aluminum alloy was designed to resist contamination and corrosion, showing how multilevel texture can protect the surface state over time [67]. Similarly, a spider-inspired aluminum alloy with microscale hierarchical structures was reported with superhydrophobic durability and reduced drag, emphasizing that multi-scale textures can support both fluid interaction control and mechanical robustness [68]. It is also important to stress that fabrication routes (laser texturing, etching, deposition, and hybrid methods) strongly influence long-term performance, because damage at one scale can break the intended wetting state even if other scales remain intact [69,70].

Hierarchical design is also useful when transport includes not only droplets on top of a surface but also fluid, cells, or molecules moving through a structured porous network. Bioinspired hierarchical carbon structures proposed as scaffolds highlight how pore hierarchy can shape wetting, capillary uptake, and mass transport while also providing mechanical support for tissue-related uses [71]. Even when the primary goal is not wetting, work on biomimetic lattice structures shows a transferable idea that hierarchy and architecture can be tuned to balance competing targets such as stiffness, deformation, and energy absorption, and the same mindset can be applied to transport surfaces where robustness, reusability, and stable anisotropy must be balanced [72].

## 4. Fabrication and Materials

### 4.1. Micro/Nanofabrication

Micro/nanofabrication provides the geometric starting point for biomimetic anisotropy such as grooves, wedges, ratchets, tilted posts, re-entrant overhangs, and hierarchical micro–nano textures that bias wetting, capillary flow, or interfacial friction. A practical theme across fabrication routes is the tradeoff between deterministic placement/order and the ability to realize more complex 3D or multiscale geometries over large areas [73]. Lithography-based patterning remains the most versatile master-making approach for anisotropic surfaces because it can prescribe micro/nanoscale features in a design-driven way. Photolithography transfers mask-defined patterns into photoresists and then into substrates, and its resolution is fundamentally tied to exposure wavelength and optics. Multiple variants (e.g., deep UV, phase shift, and interference lithography) are used to push feature size and periodicity limits.

For anisotropic transport studies, lithography is often paired with reactive ion etching (RIE) or deep reactive ion etching (DRIE) to create vertical or high-aspect-ratio structures and to translate 2D patterns into functional topography. A recurring bottleneck occurs because high-precision top-down fabrication can become tedious and expensive when each sample must be patterned and developed separately. In the context of hierarchical liquid transport surfaces, repeated sample-by-sample micro/nanopatterning makes the process costly, motivating scalable alternatives that reuse a template (e.g., template stripping) rather than rebuilding the pattern each time [74]. In this implementation, a silicon-based template is prepared with a silicon nitride film and patterned via RIE before subsequent steps generate the targeted microstructures.

Molding, embossing, and imprint-based replication address scale and throughput by separating creation of a high-fidelity master from repeated, lower-cost replication into polymers or other materials. Replica molding (RM), often treated as part of the soft lithography family, uses a soft/flexible negative and can replicate features spanning nm to mm scales, enabling a wider range of replica geometries and materials than pressure-driven nanoimprint in many cases. A concrete example is UV-based replication using elastomeric stamps. Senn et al. describe transferring a stamp pattern into a UV-curable resist via UV casting, producing replicas on polymer substrates and reporting successful reproduction down to ~50 nm feature sizes, illustrating how imprint/replication workflows can carry nanoscale details into practical materials [75].

For anisotropic transport, hierarchical groove textures are frequently produced by combining a micro-groove architecture with a secondary nanoscale roughness or re-entrant geometry. Kang et al. used a UV-assisted micromolding route (with nanoparticle-containing UV-curable precursors) to fabricate dual-scale anisotropic groove microstructures, comparing prism, rectangular, and re-entrant/overhang designs to tune directional oil sliding behavior [76]. Their fabrication workflow explicitly includes replica molding and post-processing (e.g., UV–ozone exposure to expose nanoparticles and selective removal of polymer resin), showing how microfabrication plus controlled texture activation can yield robust hierarchical anisotropy.

Etching-based fabrication is particularly powerful when the substrate crystallography can be leveraged to create well-defined anisotropic facets. In silicon, anisotropic wet etching in KOH or TMAH produces crystallographically governed profiles. Berenschot et al. use this principle in a recursive scheme to generate 3D fractal structures in single-crystalline silicon [75]. The approach can drive feature sizes down substantially (e.g., reported minimum cavity sizes on the order of hundreds of nanometers after multiple iterations) and produce fine pores below ~100 nm, which is relevant when directional transport depends on multiscale capillarity and pinning.

Laser micro/nanofabrication offers a complementary path that is maskless and programmable, and can be attractive for rapid prototyping or for patterning where conventional lithography is inconvenient. Chen et al. report femtosecond laser spatial shaping with continuous scanning (using an SLM-based beam-shaping approach) to create anisotropic microstructures for directional liquid transport, emphasizing controllable modulation of surface morphology and energy-density gradients [77]. Their results include rapid film motion along fabricated gradients (e.g., millimeter-scale transport within sub-second times) and explicitly position the method as an alternative to mask-based lithography for gradient, directional structures

### 4.2. Additive Manufacturing

Additive manufacturing (AM) is attractive for directional transport because it couples geometry, composition, and processing in a single, programmable workflow. In particular, AM can align and spatially pattern fillers while also controlling overall shape and composition, which is useful when transport depends on directional roughness, asymmetric capillary pathways, or anisotropic wettability [78].

Vat photopolymerization in the form of Stereolithography (SLA and Digital Light Processing (DLP) is a simple approach for printing microstructures [79]. SLA/DLP can encode anisotropy through designed micro-topography and field- or flow-assisted filler orientation during curing. A clear trend in recent work is to combine stereolithography with external stimuli such as acoustic fields, ultrasound-directed assembly, or shear during printing to bias particle/fiber orientation and thereby program directional mechanical or surface responses [80]. Beyond single-resin prints, vat photopolymerization also supports gradient and multi-material architectures by controlling crosslink density (via exposure time/intensity) and/or exchanging resin formulations during printing.

These strategies matter for transport because they allow one to place hydrophilic/hydrophobic domains, stiffness gradients, or porosity contrasts exactly where they can create a Laplace pressure bias, pinning asymmetry, or preferential flow route. At the same time, multi-material routes introduce practical constraints such as cleaning between resins and interfacial stress/adhesion issues that can limit robustness if the transport function relies on a sharp interface.

Two-photon polymerization (TPP) is valuable when the transport mechanism depends on micro/nanoscale features. For example, contact-line pinning asymmetry or capillary diodes at very small length scales can be achieved using TPP. However, a common bottleneck occurs because multi-material AM is typically limited to coarser scales (often on the order of hundreds of micrometers), which conflicts with the high-resolution needs of many anisotropic surface designs [80]. One hybrid solution is acoustic streaming-assisted TPP, where nanoparticles are first patterned by acoustic streaming and then locked in by localized photopolymerization. Lichade and Pan demonstrate this as a layer-by-layer workflow that produces anisotropic multi-material surfaces at micro/nanoscale [78]. The same study reports a functional transport-relevant outcome. Grooved nanoparticle-patterned samples showed substantially improved water collection performance (reported as ~3× higher efficiency versus an isotropic, flat control), consistent with the idea that engineered anisotropy can amplify capture/transport by stabilizing preferred flow paths and wetting states.

Hybrid printing at larger scales using direct ink writing (DIW) and structural anisotropy from layer-wise builds is another approach for AM of anisotropy. For applications where transport is governed more by mesoscale tracks/channels than nanoscale pinning, extrusion-based printing such as DIW can be sufficient and often easier to scale. For example, DIW-printed mesh membranes can be designed so that grooves act as tracks that guide droplets along prescribed directions. Sun et al. explicitly frame the groove structure as a guiding track that enables directional droplet transport [81]. They also emphasize that directional transport can be achieved by tuning mesh structure parameters through DIW, without extra post-processing such as surface modification.

Finally, even when chemistry is uniform, AM can generate transport anisotropy via layer-induced microstructures. In selective laser sintering (SLS), Wu et al. attribute internal anisotropic water transport to anisotropic structures formed between printed layers and within-layer pores, and show that adding hydrophilic particles (glass beads) can create long capillary channels that reinforce this directionality [82].

### 4.3. Surface Modification and Coatings

Surface micro/nanogeometries are often the skeleton of anisotropic transport, but coatings and surface modification provide the interface control that determines whether a liquid actually depins, slides, or wicks in a predictable direction. In practice, directional transport performance is highly sensitive to contact-line pinning and contact angle hysteresis, which are governed by the combined effects of surface chemistry (surface energy, polarity, functional groups) and texture (multi-scale roughness, porosity, and re-entrant features) [83]. A common strategy is wettability patterning by creating spatially programmed wetting contrasts such as hydrophilic tracks on hydrophobic backgrounds and/or gradients that bias spreading/imbibition and stabilize one-way flow paths. Such chemical asymmetry can be introduced through approaches such as chemical vapor deposition, UV/photochemical treatments, and photolithographic patterning, often combined with porous substrates or hierarchical textures to amplify capillary effects.

A concrete example is spatial wettability patterning in thin porous materials, which enables multiple 3D transport routing modes (transport on the surface vs. through-thickness vs. coupled pathways) by selectively controlling where the liquid is allowed to penetrate and where it is blocked [84]. For droplet motion on textured solids, coatings are frequently used to decouple chemical uniformity from morphological heterogeneity. This matters because strongly hydrophilic domains can trap droplets and increase adhesion, limiting long-range transport even if a pattern can guide motion. One solution is to keep the surface chemistry largely homogeneous (minimizing high-energy patches) while using patterned roughness to create regions of high apparent contact angle with low hysteresis. This approach enabled continuous, controllable directional water transport on hydrophobic/superhydrophobic patterned surfaces in a coating-based fabrication route [85].

Coating-enabled switching and scalable deposition methods also show up as practical methods through which to engineer domain connectivity and droplet pathways. For instance, a hydrophobicity-triggered transition strategy was used to evolve patterns from isolated to connected superhydrophobic domains, explicitly leveraging the idea that wettability is set by both intrinsic chemistry and surface structure, while electrospraying was highlighted as a flexible method with which to control morphology and surface chemistry during fabrication [86].

A particularly powerful coating paradigm for reducing pinning is SLIPS/SLIMS (slippery liquid-infused porous/microstructured surfaces), where a lubricant phase forms a stable, low-shear interface that suppresses contact-line trapping. Low-adhesion, lubricant-infused surfaces are frequently positioned as enabling long-distance, rapid transport because they combine stable apparent contact angles with minimal resistance to droplet motion, and transport can be tuned by changing either the surface structure or the physicochemical properties of the infused lubricant [87]. In an example directly relevant to anisotropy, slippery liquid-infused microstructure surfaces were fabricated by laser texturing followed by fluoroalkylsilane treatment (to reduce surface energy and stabilize the lubricant) and oil infusion/spin-coating. Importantly, rice-leaf-like anisotropic grooves produced direction-dependent differences in transport distance, consistent with a mechanism that balances Laplace pressure driving against direction-dependent adhesive forces [87].

Beyond mobility, coatings and lubricant layers are also used to improve contamination tolerance (a practical form of antifouling in transport contexts). Directional oil-sliding surfaces, for example, were explicitly motivated not only by transport/collection of residual oils but also by preventing contamination from oils in applications spanning microfluidics and industrial handling. More broadly, lubricant-infused slippery platforms are discussed as bioanalytical enablers precisely because they reduce hysteresis and pinning conditions that otherwise promote residue retention and inconsistent sample handling. Lubricant-infused slippery ratchet arrays, for instance, have been used to achieve large-volume, fast omni-droplet transport for biomedical detection workflows.

### 4.4. Soft and Responsive Materials

Soft and responsive materials add a second layer to biomimetic anisotropy. Geometry (grooves, cilia, ratchets, microarrays) sets the preferred direction and the local force asymmetry, while the material response (swelling, programmed strain, magnetic bending, shape-memory recovery, or wettability switching) sets when that asymmetry is active, how fast it operates, and how robustly it survives real environments. In practice, the most useful systems are those where mechanical compliance and stimulus response are co-designed with anisotropic topography so that transport directionality is preserved even as the surface deforms, fouls, or cycles through actuation.

Hydrogels are a natural choice for directional transport because they can generate large, reversible shape changes at low stress and can be patterned so that deformation is intrinsically anisotropic. A clear example is the 3D printing of anisotropic hydrogel actuators in which patterning and material selection produce direction-dependent swelling and motion, enabling bioinspired transformations and locomotion-like behaviors [88]. A key point for transport is that response speed can become a design variable. Introducing internal microchannels for water transport can accelerate actuation, while anisotropic hydrogel architectures can yield markedly different volumetric responses along different directions (e.g., large vs. small volumetric change depending on how the responsive layer is patterned/combined). For anisotropic transport of liquids/soft matter, these hydrogel-based strategies are most compelling when swelling-induced curvature gradients or cyclic deformation is coupled to an asymmetric contact-line condition (pinning/hysteresis asymmetry) or to a cilia-like stroke that biases flow.

Liquid–crystal elastomers (LCEs) and liquid–crystal networks (LCNs) provide a powerful route to programmable anisotropic deformation because the director field encodes a preferred strain direction. In printed artificial cilia based on LCN actuators, the surface is fabricated so that light triggers bending. Arrays can then create cilia-like motion that is relevant for pumping and mixing in wet environments [89]. The underlying fabrication concept is also important for this approach. Self-organizing inks and alignment control allow actuation directionality to be written into the material during processing, and the actuator can be driven modularly by light. For transport-of-matter applications, the main advantage of LCE/LCN systems is that they can generate repeatable, spatially varying deformation fields (bending/twisting/waves) without hard mechanical linkages, which makes them well matched to microstructured anisotropic surfaces where fragile features would otherwise break.

A widely used strategy for responsive anisotropic transport is to embed magnetic particles in a soft polymer matrix to form field-driven cilia that generate non-reciprocal strokes and metachronal waves. PDMS prepolymer is commonly used as the matrix due to its soft/tunable mechanics and processability, while magnetic particles (e.g., iron oxide nanoparticles or carbonyl iron microparticles) set the magneto-mechanical response [90,91]. In this method, solvent adjustment can be needed to manage composite viscosity during molding, and that magnetization direction and particle distribution can be programmed during curing to encode bending direction and actuation phase relationships across arrays. A translational example in a realistic transport setting is a ciliary airway stent concept, where magnetic artificial cilia are built as a multilayer soft composite (magnetic NdFeB–silicone rubber layer encapsulated by PDMS layers), and microstructuring increases surface area to support a robust hydrogel coating for wetting-assisted pumping [92]. In this example, transport directionality is linked to two coupled asymmetries which are an encoded antiplectic metachronal wave and a programmed non-reciprocal power/recovery stroke, plus surface property tuning (hydrophilic vs. hydrophobic) to improve pumping of viscous mucus-like fluids [92].

A limitation of soft microstructured transport surfaces is mechanical fragility. One solution is to generate robustness through an interplay of materials and architecture. For example, robust superhydrophobic smart cilia can be fabricated by aligning iron-laden PDMS aerosols under a magnetic field to form microwires that can be hidden/protected and then re-erected for operation, followed by nanoparticle-based superhydrophobization [93]. Here, the core design goal is explicitly framed as maintaining droplet impact/adhesion/transport functionality after abrasion and environmental exposure, which makes responsive soft microstructures deployable outside ideal lab conditions.

Beyond mechanical actuation, responsive polymers can switch surface energy and therefore switch transport regimes on anisotropic textures. A representative case uses temperature-responsive PNIPAAm-based surfaces, where wettability changes across the LCST because hydrogen bonding and polymer conformation change, producing hydrophilic-to-hydrophobic transitions and swelling/shrinkage effects [94]. When such chemistry is combined with an anisotropic microarray, the net result is not just more or less wetting but the ability to switch the direction of liquid transport by toggling the stimulus.

Shape memory polymers SMPs extend the design from continuous actuation to shape programming and recovery, which is useful when transport anisotropy should be toggled between discrete modes such as stored/flat vs. deployed/textured [95,96,97,98]. In the context of printed anisotropic polymers, multi-material approaches are highlighted as a practical route to combine different functions, i.e., SMPs are integrated with other materials to add mechanical strength, conductivity, or magnetic functionality. For transport-of-matter surfaces, the opportunity is to build reconfigurable anisotropic textures that can be deployed on demand (or self-deployed under heat/light), while still retaining adequate stiffness, fatigue resistance, and environmental stability.

## 5. Transport Mechanisms in Biomimetic Anisotropic Systems

This section explains how biomimetic anisotropy is converted into directional transport by coupling a driving bias with direction-dependent resistance. Across the systems reviewed here, rectification usually emerges from the balance between capillary driving forces, retention and depinning thresholds. Presenting these mechanisms in a purely narrative sequence can make it difficult for readers to identify which strategy is most suitable for a given operating regime (droplets versus films, along-surface versus through-thickness transport, liquids versus particles/soft objects) and for real-world constraints such as contamination, abrasion, or wetting state transitions. To make this section more directly actionable, Table 1 provides a concise comparative mechanism-to-design toolbox that organizes the major anisotropic transport mechanisms by operating regime, dominant driving forces, key design knobs, robustness considerations, and recommended metrics for cross-study comparison.

### 5.1. Directional Wetting via Contact-Line Pinning Asymmetry

Directional wetting can be created when the three-phase contact line meets different pinning barriers depending on the direction it tries to move. The main principle is simple. If advancing (or receding) is easy in one direction but strongly hindered in the opposite direction, the liquid front will preferentially propagate one way and stick the other way. On asymmetric textures, this shows up as direction-dependent contact angles and direction-dependent contact line motion, which together act like a wetting diode. This was demonstrated clearly by designing asymmetric nanopillar arrays where a deposited liquid propagated in one preferred direction while pinning in other directions [99].

At the micro/nanoscale, the asymmetry does not need to be a macroscopic slope or a large free-energy gradient. Instead, the asymmetry is embedded in the geometry that the contact line must interact with. In the mentioned example, asymmetric nanopillars introduce directional energy barriers [99]. Thus, the contact line can advance by finding lower-cost pathways in one direction, while the reverse direction requires overcoming higher barriers, so the front pins. In practice, this means one can tune directionality using structural parameters (for example, the degree of asymmetry and spacing/height ratios), while the intrinsic wettability still matters because it sets how strongly capillarity pushes the contact line against those barriers.

A closely related ratchet picture is the pin release mechanism. The contact line repeatedly pins on a feature, builds up a driving force (via deformation and increasing local contact angle/curvature), and then releases once a threshold is exceeded, but the threshold differs by direction when the roughness is asymmetric. Malvadkar et al. reported an engineered nanofilm of tilted polymer nanorods that shows anisotropic wetting by means of a pin-release droplet ratchet mechanism, producing markedly different retention forces in the pinning vs release directions [100]. They quantified this anisotropy via larger critical drop volumes and higher retention forces in the pinning direction, reaching differences on the order of tens of micronewtons. The key transport implication is that once you provide any periodic forcing (tilt, vibration, or controlled perturbations), the drop will preferentially step in the low-barrier direction while resisting motion in the opposite direction.

This direction-dependent pinning is also why multiscale textures can either help or hurt, depending on how they change the fraction of the contact line that is actually pinned at each scale. Paxson and Varanasi directly imaged contact-line depinning on textured surfaces and showed that adhesion and hysteresis are governed by microscale pinning/deformations and by capillary bridges at the receding contact line [101]. They reported that hierarchical roughness can reduce or increase pinning and proposed a model where reducing adhesion requires the product of pinned fractions across length scales to be sufficiently small. For designing directional wetting surfaces, this matters because adding more texture is not automatically beneficial. The added scale must preserve the intended directional asymmetry without introducing new symmetric pinning sites that trap the contact line in all directions.

Although pinning is often discussed as a geometric effect, the same asymmetry can be created or amplified by chemical heterogeneity at the contact line. Molecular-level work shows how a pinned contact line can still adjust its pinning force in response to changing driving forces, even when the apparent contact line position is fixed. Huang et al. describe pinning force as a key factor controlling droplet dynamics and connect self-adaptive pinning to local molecular asymmetry on chemically heterogeneous substrates, proposing a model that links pinning force to asymmetry in the local distribution of liquid atoms [102]. This micro-to-macro view is useful when you combine texture with patterned chemistry. The macro-directionality you observe can be the cumulative result of many local asymmetric pinning events.

Finally, even when the intended mechanism is a wettability contrast (rather than a purely ratcheted texture), directional motion still appears through unbalanced forces acting on the three-phase contact line. Yuan et al. show that asymmetric spreading and directional retraction toward a more hydrophilic region arise from unbalanced net forces along the contact line, and they discuss a Young’s force form tied to differences in local contact angles [103]. In real surfaces, contact-line pinning and hysteresis set the threshold that this imbalance must overcome, which is why ideal gradient-driven motion can fail (stick–slip or complete arrest) if pinning barriers are too strong.

### 5.2. Capillary Pressure Bias from Curvature and Wedge Geometry

A second major route to rectified transport is to build a capillary pressure gradient into the geometry itself. When a liquid interface is constrained by an anisotropic shape (e.g., a tapering wedge, a conical spine, or a groove with a changing cross-section), the local meniscus curvature varies along the structure. By the Young–Laplace relation, the pressure jump across the interface scales with curvature, so spatial variation in curvature becomes a pressure field that can bias flow and droplet motion even on chemically uniform materials [104]. In bioinspired designs, conical and wedge-like morphologies provide a clear illustration of how curvature gradients translate into directional transport. For example, in cactus-spine-like geometries, the Laplace pressure difference along a taper can be expressed in terms of the curvature at two positions (e.g., radii $r_{1}$ and $r_{2}$), creating a pressure bias that drives droplet migration along the spine [105].

More broadly, conical microstructures and spindle–joint morphologies (e.g., spider silk) are recurring natural motifs where a curvature difference between adjacent regions sustains a Laplace pressure imbalance that promotes net liquid relocation [105]. Importantly, the preferred direction can depend on wettability and whether the curvature gradient is imposed on external features (convex) versus internal cavities (concave). In conical tubes, for instance, the reported direction reverses with surface chemistry. Droplets tend to move from the wider region toward the narrow region on hydrophilic inner surfaces, while the opposite direction can occur on hydrophobic inner surfaces.

Engineered capillary diodes and wedge tracks translate these principles into practical transport elements. A representative open microfluidic implementation uses wedge-patterned, wettability-confined tracks where geometry and boundary conditions collectively generate a net capillary driving force. In a widely used approach, superhydrophilic tracks are patterned within a superhydrophobic background so the contact line is laterally confined while the axial width changes. Liquid dispensed near the narrow end can be transported toward the wider end through a combination of hemiwicking and Laplace pressure-driven flow [106]. In that study, the capillary force increased approximately linearly with wedge angle, and a wedge of ~3° produced peak forces on the order of ~56 μN. Transport speeds were tunable and reached ~110 mm/s on dry tracks and ~300 mm/s on pre-suffused tracks, with reported peak flow rates up to ~350 μL/s and velocities exceeding ~400 mm/s under optimized conditions. These results highlight a useful design rule for wedge-based rectification: increasing the geometric asymmetry (e.g., wedge angle) can increase the available capillary driving force, but joint losses and pinning can still limit long-range transport unless the diode is engineered to manage meniscus transitions and dissipation.

Recent work also shows how additive manufacturing enables modular applications of wedge-driven Laplace biases at larger (mm–cm) scales while keeping the same underlying mechanism. A 3D-printed platform composed of sequential wedge notches and joint grooves was explicitly framed as using an asymmetric-structure-induced Laplace pressure differential as a passive driving strategy [107]. By introducing crescent-shaped grooves at unit joints, the platform converted accumulated potential energy at joints into kinetic energy and added an additional Laplace contribution at the connection, enabling a reported maximum velocity of ~18 mm/s and transport distances up to ~350 mm with 14 wedge units in series. This serial wedge and joint management concept is instructive for scaling. Long-distance performance is not only about maintaining a curvature gradient within a unit but also about minimizing or actively compensating the hydraulic/meniscus penalties at repeating interfaces between units. More generally, structural capillarity frameworks formalize these ideas by treating geometric heterogeneity as a programmable way to generate spatially varying average curvature and therefore Laplace pressure gradients that drive liquid migration in open microfluidic settings.

Finally, curvature-induced pressure biases can also appear in dynamic regimes where the interface is continually reshaped by external forcing. On heated concentric microgroove arrays, for instance, droplet motion and rebound direction were linked to a Laplace pressure difference between inner and outer boundaries of the grooved geometry, illustrating that even when inertia and phase change matter, the geometric Laplace imbalance can still provide a directional bias [108].

### 5.3. Soft Anisotropy: Elastocapillarity and Compliance-Driven Rectification

Soft, deformable anisotropic structures provide another method of control over transport beyond geometry and surface chemistry, that is, mechanics. When an interface is compliant, the solid can deform under capillary stresses, gravity, or external fields, and that deformation can be direction-dependent such as bending of tilted hairs, asymmetric collapse of grooves, or strain-induced reshaping of a mesh. The result is a mechanical asymmetry that changes the local contact state, friction/adhesion, and hydraulic resistance in a way that can bias transport in one direction over the other. One clear manifestation is stiffness-guided droplet motion that is often discussed as droplet durotaxis, where a droplet on a substrate with a Young’s modulus gradient tends to move toward the softer region because surface-tension-induced deformation near the contact line becomes asymmetric across the drop footprint.

Elastocapillarity becomes important when capillary forces are large enough to bend or reorganize slender features (pillars, hairs, lamellae) or to locally compact porous/soft regions. In engineered micro/nanostructures, this is sometimes exploited intentionally as a post-fabrication reconfiguration route where capillary forces can drive densification and self-assembly of high-aspect-ratio arrays (e.g., elastocapillary aggregation), which can transform an initially symmetric array into directionally biased clusters or channels [73].

From a transport perspective, the key point is that capillarity does not merely pull liquid along but can reshape the pathway itself, changing the effective curvature, local confinement, and flow resistance in a history- and direction-dependent manner. A recent example that makes this coupling explicit is the metatongue concept, which is an open, grooved, deformable sheet that wicks liquid and then undergoes elastocapillary deformation so that grooves close during imbibition/handling. In this design, capillary forces drive sequential uptake, while poroelastic/elastocapillary deformation provides on-demand confinement (groove closure), enabling fast transport and controlled retention/compartmentalization of liquid [109]. The mechanism is inherently rectifying. The structure presents a more open geometry for rapid uptake, then transitions toward a more confined configuration that stabilizes the liquid by reducing slosh-back or redistributing where the liquid resides along the grooves. Conceptually, this illustrates how compliant anisotropic microstructures can act as mechanical valves for capillarity when geometry is not fixed, so the capillary pathway can be programmed to switch as wetting proceeds.

Compliance-driven rectification is also prominent in hair/cilia-like arrays, where directional bending changes contact area and dissipation. In robust smart superhydrophobic cilia, the lateral adhesion force is anisotropic. Motion parallel to the cilia direction exhibits a larger adhesion force than motion perpendicular to it, and this anisotropy is attributed to elastic deformation of the microwires during contact and depinning [93]. This kind of mechanical anisotropy is especially powerful under periodic forcing like vibration, because even when the external input is symmetric in time, the contact-line response is not. The deformable cilia create different pinning/depinning thresholds depending on direction and thus can bias net motion over cycles.

Related strategies combine compliance with programmable actuation. Heteromorphic magnetically steerable interfaces that integrate a heterogeneous elastic modulus/wettability configuration (rigid hydrophilic cilia on a soft hydrophobic base) substantially increase bubble pinning and enable controlled bubble transport by adjusting magnetic inclination and applying vibrations [110]. These examples highlight a broader design rule that the combination of tilt, flexibility, and directional loading yields different deformation states for forward vs. backward motion, which translates into asymmetric adhesion and effective resistance.

Finally, soft anisotropy can be made reconfigurable through macroscopic deformation of the substrate. Three-dimensional-printed stretchable elastomeric meshes provide anisotropic wettability that can be tuned by tensile strain (by changing mesh aperture and the droplet–mesh contact geometry), and bending further alters droplet adhesion and sliding angles [111]. In transport terms, strain/bending lets a single surface undergo anisotropy on demand, which is useful when the same platform must alternately promote rapid spreading (low resistance) or promote retention/pinning (high resistance) along selected directions.

### 5.4. Active Rectification Under Oscillatory Forcing

A common active rectification strategy is to apply a zero-mean oscillatory input like sinusoidal vibration, acoustic excitation, or periodic electromechanical actuation and rely on spatial anisotropy to break symmetry so the system produces a net drift over each cycle. In droplet and film systems, the rectification typically comes from cycle-by-cycle asymmetry in depinning thresholds at the contact line, capillary/Young force imbalances during deformation, or direction-dependent viscous resistance created by anisotropic topography.

A clear example is the anisotropic ratchet conveyor (ARC) concept, where purely vertical harmonic vibration is converted into horizontal droplet motion by an anisotropic surface geometry. Within this concept, the droplet experiences repeated contact-line oscillations, but the pinning/depinning conditions differ in the forward versus backward direction, so slip happens preferentially in one half of the cycle, yielding a finite displacement per period [112]. This mechanism is often described as a pin release ratchet. Vibration increases and decreases the effective normal load and interfacial forces over a cycle, but the asymmetric microgeometry makes the net depinning probability (or depinning threshold) direction-dependent. A practical implication is that ARC-style transport frequently exhibits a threshold in forcing amplitude (or acceleration) below which the contact line remains pinned and the net drift vanishes and above which directed motion emerges.

Importantly, once the basic rectification element exists, additional microstructural design can turn transport into function. Patterned tracks, junctions, and features can enable operations such as guiding, gating, and routing on a vibrated substrate [113]. One practical limitation of open vibrating droplet systems is evaporation. Enclosing the droplet while still using vibration-driven rectification has been explored as a method through which to retain the actuation principle while improving robustness for longer operation times [114].

Beyond ARC-type discrete ratchets, several studies show that asymmetric textures alone can bias vibration-driven droplet motion. For instance, asymmetric structured surfaces can induce directional motion once a forcing threshold is exceeded, with motion governed by a competition between the driving and capillary retention forces and by how the texture scales relative to the droplet and the vibration [115].

Mesoscale and microscale asymmetries can be combined with chemical patterning to strengthen rectification. For example, dissipative particle dynamics simulations demonstrate directed transport on vibrating substrates that combine asymmetric corrugations with patterned wettability and show that design choices (including vibration period and wettability layout) can strongly affect both transport distance and collective behaviors such as bringing multiple droplets together [116]. It is also useful to separate anisotropy-enabled rectification from rectification that arises from intrinsically nonlinear droplet dynamics under vibration. Even on simpler (non-ratcheted) settings, net drift can appear when vibration excites coupled droplet modes so that the cycle-averaged horizontal Young force contribution does not cancel. A quantitative study of vibrated sessile drops shows that a net displacement per cycle can be explained by unbalanced Young forces integrated over the oscillation and that directed motion can require the presence and coupling of distinct deformation modes (e.g., rocking and pumping) [117].

In a biomimetic design context, engineered anisotropy is attractive because it offers geometric control over directionality and can reduce reliance on delicate mode selection conditions. Acoustic and electromechanical actuation fit naturally into this framework because they provide controllable oscillatory forcing. For example, droplet transport can be driven by ultrasonic surface waves, by mechanical vibration, or by acoustic radiation pressure, all of which are oscillatory inputs that can be rectified by suitable anisotropic boundary conditions or textures.

On the solid transport side, vibration-driven stick–slip platforms provide a close mechanical analogue of ratcheting: by alternating between sticking (static friction) and slipping (kinetic friction) under a periodic excitation, a net step can be produced per cycle, and this can be scaled to parallel manipulation strategies [118].

## 6. Applications of Anisotropic Sample Transport

### 6.1. Pump-Free Microfluidics and Point-of-Care Sampling

A straightforward and widely explored application area for anisotropic transport is passive, pump-free liquid handling by moving, routing, and conditioning small volumes using geometry (Laplace pressure differentials, preferential wetting pathways, or joint “diodes”). For example, paper/fabric-based microfluidic bandages can autonomously pull and route biofluids through embedded capillary networks, enabling wearable sampling concepts that are compatible with low-resource diagnostics. Related open architectures extend this idea to reconfigurable capillary frameworks, where anisotropic unit geometries (e.g., wedge/corner elements) and network design encode directionality, mixing points, and flow-path logic. Nature-inspired rectifiers are also being translated into device-like fluidic components. A recent example explicitly positions a hummingbird-tongue-inspired design strategy as a route to capillary liquid diodes that can support point-of-care diagnostic tasks [109].

**Figure 9:**
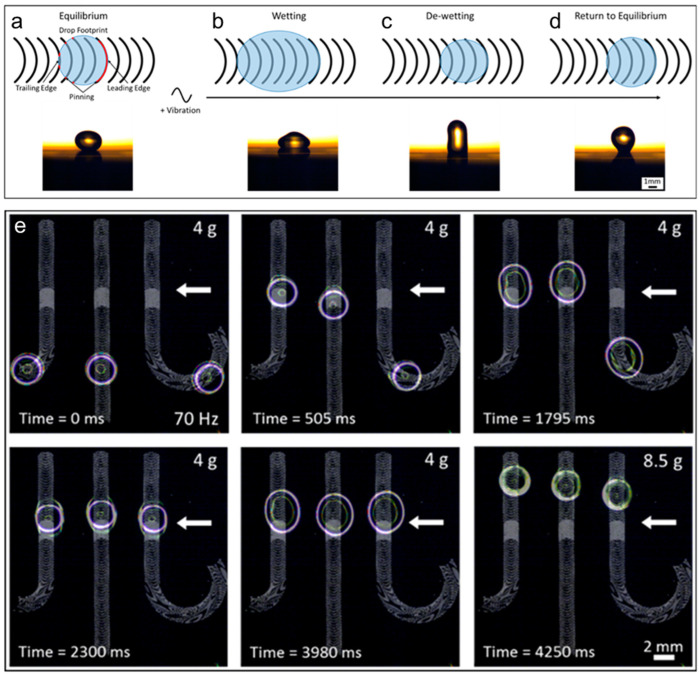
Anisotropic ratchet conveyor (ARC) droplet transport and gate-enabled synchronization. (**a**–**d**) ARC systems transport droplets through an anisotropic surface pattern composed of periodically occurring curved rungs (black) defined by a hydrophobic background (white). This asymmetric geometry creates a difference in pinning between the leading and trailing edges of the contact line (droplet footprint). Applied orthogonal vibrations drive the contact line through wetting, de-wetting, and equilibrium states, producing a net droplet step per vibration cycle and thus directed transport. (**e**) Droplet synchronization with ARC gates: droplets transported on distinct ARC paths under vibrations below the gate threshold pause at a duty-cycle transition (16.6% to 8.3%) and remain pinned until the vibration signal is increased above the gate threshold, after which transport resumes in a tight distribution. Reproduced from [113] under the Creative Commons Attribution (CC BY 4.0) license.

Figure 9 highlights a complementary low-infrastructure route to droplet handling in pump-free microfluidics; this is through anisotropic ratchet conveyors, where a passive surface pattern is paired with a single external vibration input to drive discrete droplets along predefined tracks [113,114]. The curved-rung geometry biases contact-line pinning so that each oscillation cycle yields a net step, enabling transport and routing without embedded pumps or valves. Beyond transport, ARC gates provide timing control by pausing droplets at a designed duty-cycle transition and restarting motion by increasing the vibration amplitude, which enables synchronization, staged delivery to detection zones, and protocol-like sequencing for point-of-care workflows.

### 6.2. Long-Distance Transport, Routing, and Handling for Engineered Systems

Beyond short-range droplet motion, anisotropic designs are increasingly framed as enabling long-distance spontaneous transport with controllable trajectories, which is attractive for practical integration (reduced tubing, fewer pumps/valves, simpler maintenance) [119,120,121,122]. For example, a modular wedge-based platform fabricated by 3D printing and surface modification is described as being applicable to microfluidics, fuel cells, phase-change heat transfer, and water harvesting, while also supporting functions like diversion and splitting in a pump-free manner [107]. The same work explicitly highlights path-customizable transport as an available solution for biosensing and fuel-cell contexts. This is a useful example of how biomimetic anisotropy can be packaged into an engineering transport module rather than a single-purpose surface.

**Figure 10:**
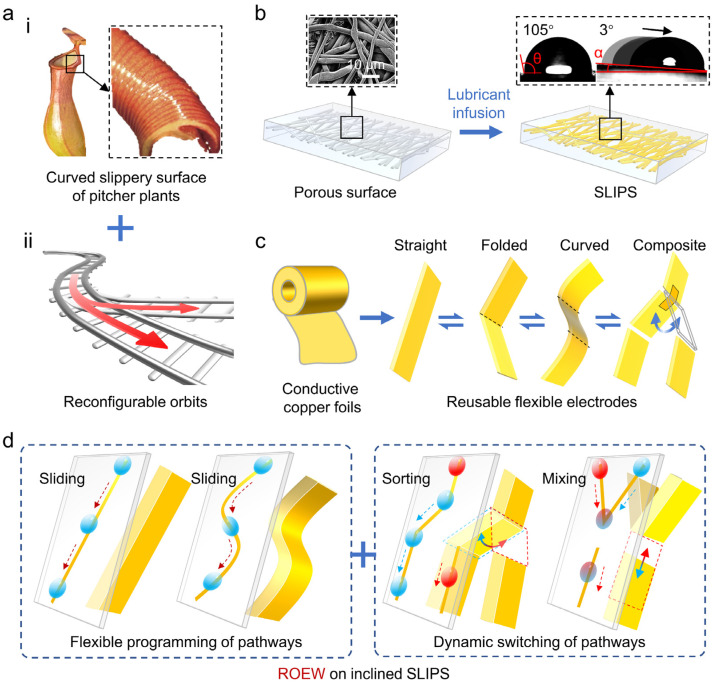
Reconfigurable orbital electrowetting (ROEW) on inclined slippery liquid-infused porous surfaces (SLIPS) as a programmable droplet-routing and handling module. (**a**) Design inspiration for SLIPS (pitcher-plant-inspired slippery interface) and the ROEW concept based on reconfigurable orbital electrode geometries. (**b**) SLIPS preparation and characterization, including porous substrate microstructure, hydrophobic wetting state, and low-adhesion/superlubricity behavior. (**c**) Reusable flexible electrodes that can be cyclically deformed into straight, folded, curved, and composite shapes to reprogram transport pathways. (**d**) Demonstration of versatile droplet manipulation on inclined SLIPS by dynamically reconfiguring non-contact electrodes. Reproduced from [123], CC BY 4.0.

A complementary direction is to pair low-adhesion transport layers with reconfigurable routing logic so that long-distance motion is not only possible but also programmable. For example, reconfigurable orbital electrowetting on inclined slippery liquid-infused porous surfaces uses non-contact, deformable electrodes to print and erase wettability pathways in real time, enabling continuous droplet transport along routes that can be re-shaped from straight lines to 90° turns and S-shaped bends on the same substrate (Figure 10) [123]. In addition to routing, the same platform supports on-demand handling functions such as online sorting and temporal mixing by dynamically switching composite electrode layouts. This type of reusable, reconfigurable routing layer illustrates how open-surface transport can be packaged into an engineered module when reduced tubing, fewer valves, and flexible trajectory control are priorities.

### 6.3. Environmental and Water Management Uses

Directional transport is widely discussed as a key enabler for atmospheric water harvesting because surfaces must nucleate/collect, move liquid against pinning and gravity constraints, and deliver it to reservoirs efficiently [124,125,126]. Nature-inspired strategies for efficient atmospheric water harvesting are becoming increasingly important, where droplet capture and guided transport are central to system-level performance [105]. At the component level, transport surfaces are also increasingly connected to thermal management needs (e.g., controlling condensate removal), aligning with the broader view that anisotropic liquid transport underpins both water/energy harvesting and heat transfer functionality [74].

**Figure 11:**
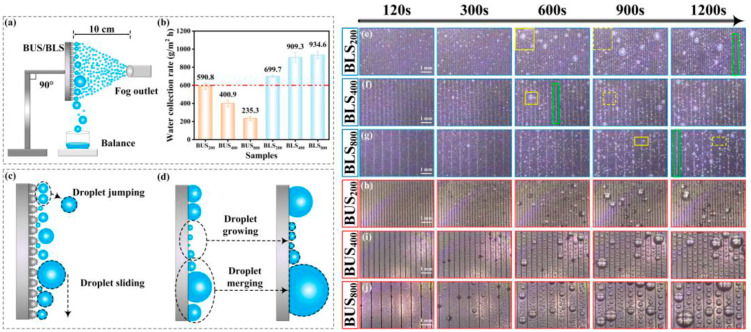
Fog collection test of Indocalamus-leaf-inspired bionic upper surfaces (BUSs) and bionic lower surfaces (BLSs). (**a**) Schematic of the homemade water/fog collection apparatus used to quantify harvesting, including sample mounting and the droplet collection container. (**b**) Fog collection efficiency (water collection rate, g/m^2^ h) for BUSs and BLSs prepared at different laser scanning intervals. (**c**) Representative droplet-removal modes on BLSs, highlighting coalescence-induced droplet jumping and gravity-induced droplet sliding. (**d**) Representative droplet behavior on BUSs, where higher adhesion delays removal until droplets grow and merge. (**e**–**j**) Time-lapse images of fog collection on BUSs and BLSs from 120 s to 1200 s. Reused from [127] licensed under CC BY 4.0.

A clear example of how anisotropic wetting and adhesion control translate into water-harvesting performance is provided by Indocalamus-leaf-inspired bionic upper surfaces (BUSs) and bionic lower surfaces (BLSs) fabricated by laser scanning plus chemical modification (Figure 11) [127]. In a controlled fog collection setup (25 °C, 90% relative humidity; fog flow rate 175 mL/h; 10 × 10 mm samples placed 10 cm from the fog outlet), the BLSs consistently outperformed the BUSs with the same laser scanning interval (SI), reaching a maximum fog collection efficiency of 934.6 g/m^2^ h on BLS800 compared with 235.3 g/m^2^ h on BUS800 (about 3.98× higher). This performance gap was linked to adhesion; the BLSs’ hierarchical micro/nanostructures produced lower adhesion, enabling rapid condensate removal through coalescence-induced droplet jumping and gravity-driven droplet sliding, which continuously refreshes the surface for further nucleation and growth. By contrast, higher adhesion on the BUSs delayed droplet detachment until droplets grew and merged enough to overcome pinning, which reduces harvesting efficiency by slowing turnover of condensation sites.

### 6.4. Self-Cleaning and Stability in Fouling-Prone Settings

One of the most useful applications of anisotropic directional structures is in self-cleaning surfaces [128,129,130]. Where anisotropic surfaces are expected to operate in dusty, biofouling, or chemically complex environments, performance is often limited less by initial directionality and more by how long that directionality survives real contamination and repeated use. This is especially clear for anisotropic slippery liquid-infused porous surfaces (SLIPS), where the working lubricant can evaporate, leak, or contaminate nearby interfaces and where cleaning commonly relies on water for hydrophilic dust but organic solvents for hydrophobic dust, which is undesirable for field use. A useful alternative direction is anisotropic solid slippery surfaces (ASSSs), which aim to preserve low-adhesion transport while removing the failure modes associated with mobile liquid lubricants.

**Figure 12:**
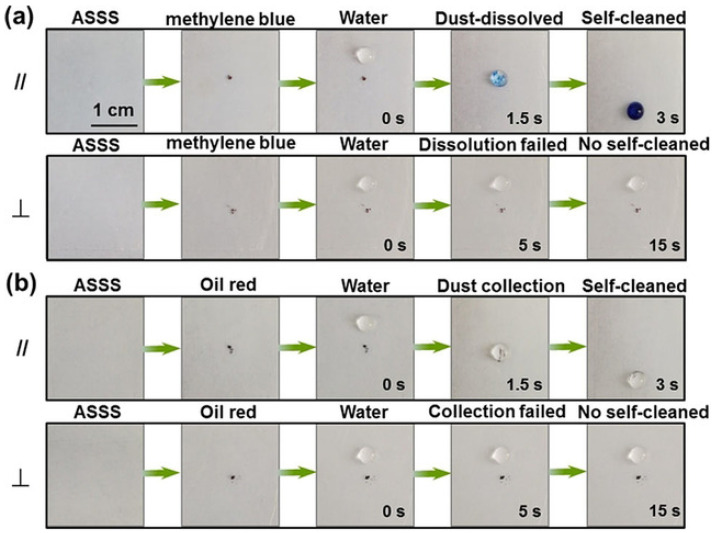
Directional self-cleaning on an anisotropic solid slippery surface with replicated micro-grooves. Time sequence photographs show a 10 μL water droplet cleaning the surface at a tilt angle of 40°. (**a**) Methylene blue (hydrophilic) powder and (**b**) oil red O (hydrophobic) powder are removed when the droplet moves parallel to the micro-grooves, but removal is not achieved in the perpendicular direction because the tilt angle is below the required sliding angle for cross-groove motion. Top rows show parallel direction (//) and bottom rows show perpendicular direction (⊥). Reproduced with permission from [131], copyright 2019 Wiley-VCH.

An illustrative example is the wax-based ASSS reported by Guo et al. [131], which combines a cellulose-rich paper substrate infused with paraffin wax (as a solid lubricant) and a replicated micro-groove array that creates direction-dependent sliding. In this design, stability is not only a material’s claim but a functional outcome: the paraffin wax does not transfer or stain other contacted surfaces, and the surface maintains droplet sliding behavior even after exposure to acidic or alkaline solutions. Durability is further supported by a restoration route that is practical for deployed systems: after mechanical damage, the surface can be reheated above the wax melting point, and the micro-grooves can be re-imprinted using a PDMS mold, returning the surface close to its initial state. Importantly for fouling-prone use, Figure 12 shows that self-cleaning itself becomes directional. At a fixed tilt angle of 40°, a 10 μL water droplet can remove both a hydrophilic contaminant (methylene blue) and a hydrophobic contaminant when motion is along the groove direction, but not when motion is perpendicular to the grooves because the tilt angle is below the required sliding angle in that direction. This kind of cleaning path anisotropy is valuable for applications because it links rectified transport to maintenance: the same geometry that routes droplets can also define preferred contaminant removal trajectories, while the solid lubricant construction reduces performance drift caused by lubricant loss or redistribution.

## 7. Outlook and Future Directions

A persistent barrier to translation is that anisotropic transport is reported using different performance metrics across subfields. Droplet studies often emphasize onset thresholds and drift speed, open capillary structures report distance and throughput, porous Janus systems focus on breakthrough pressure or one-way wicking, and solid transport is reported with frictional or stepwise motion outcomes. A practical next step is a small, shared set of metrics that can be reported across platforms. For example, a directionality ratio, a threshold condition, a robustness metric, and a contamination metric can be developed and adopted. Community challenge tests using representative liquids (water, low-surface-tension mixtures, and at least one particle- or protein-containing fluid) would make comparisons more meaningful and help to identify mechanisms that survive realistic conditions.

Many anisotropic surfaces perform well in clean demonstrations but degrade when wetting states shift or when wear, adsorption, and lubricants/surfactants alter the interface. Future designs should therefore treat robustness as part of the mechanism. Promising routes include geometry that retains directionality even when fully wetted, damage-tolerant hierarchical designs where microtextures encode direction while nanoscale features tune wettability, and renewable interfaces such as replenishable or lubricant-assisted layers that reduce friction and limit fouling. Porous and textile-like platforms can also enhance robustness through distributed pathways, but long-term cycling tests are needed to quantify failure modes such as clogging, swelling, and layer delamination.

Manufacturability is another key opportunity. While micro/nanofabrication remains valuable for mechanistic clarity, many applications require scalable, tolerant processing. Additive manufacturing is emerging as a practical bridge because it enables rapid iteration of geometry-encoded transport components and modular open architectures. The next step is to create hybrid workflows in which printed mesoscale geometry defines pathways and reservoirs, microtexturing is applied only where interfacial control is essential, and localized coatings address wetting and fouling. This approach also motivates explicit tolerance studies.

Finally, future progress will benefit from mechanism-aware design rules and simplified models that remain predictive for real liquids and soft interfaces. Soft anisotropy and elastocapillary coupling are likely to grow in importance for textiles, biomedical interfaces, and deformable architectures, while active rectification (vibration, acoustics, and magnetic/electromechanical driving) should be treated as a co-design problem where anisotropy sets direction and actuation sets the operating envelope. Across mature application areas like pump-free open microfluidics and sampling, environmental water management, porous/textile separations, and fouling-prone self-cleaning surfaces, the most impactful work will couple clear mechanisms with manufacturable designs, report shared metrics, and demonstrate robustness with realistic fluids and environmental stressors.

## Figures and Tables

**Table 1:** Comparative design toolbox for anisotropic transport mechanisms in biomimetic systems, organizing each strategy by operating regime, dominant driving forces, key design parameters, and representative use cases.

Mechanism	Operating Regime	Dominant Driving Force(s)	Key Design Controls	Best-Fit Use Cases
Directional wetting via contact-line pinning asymmetry	Sessile droplets, films; micro/nano-textured surfaces; mm–cm droplet routing	Direction-dependent depinning/retention (hysteresis) on asymmetric features (“wetting diode” behavior)	Texture asymmetry (tilt/sawtooth angle), pitch/height ratios, hierarchical texture choice, wettability/chemical patterning, expected forcing	Droplet diodes, routing on open substrates, directional self-cleaning when motion is along easy direction; threshold-controlled motion under mild forcing
Directional wetting via contact-line pinning asymmetry	Open microfluidic tracks, grooves/wedges/spines; droplet + continuous wicking; mm–cm transport elements	Spatial curvature gradient, Laplace pressure gradient (Young–Laplace) driving net flow/migration	Wedge angle/taper, groove cross-section evolution, joint design, boundary confinement, surface chemistry that can flip direction in tubes	Long-distance passive transport; structural capillarity building blocks; diode-like open routing, pump-free sampling elements
Asymmetric pores and membranes	Through-thickness transport in porous/fibrous media (Janus membranes, textiles); droplets/mixtures; filtration and separation	Direction-dependent capillary entry/barrier inside pore network (wettability contrast + pore geometry)	Wettability contrast (Janus), pore radius distribution, conical/needle-like pores, thickness and layering, surface roughness inside pores	One-way wicking, gravity-assisted separation without pumps, wearable sampling/filtration, moisture management textiles
Soft anisotropy: elastocapillarity and compliance-driven rectification	Deformable hairs/cilia/meshes/grooves; droplets + soft objects; interfaces that reconfigure during wetting/handling	Capillary forces + direction-dependent deformation (bending/closure/strain) changes contact/friction/resistance; stiffness-guided motion	Young’s modulus gradients, feature aspect ratio and tilt, groove/mesh geometry that can close/open, programmed strain/bending, coupled actuation	Textiles/soft biointerfaces, deformable open architectures, transport + retention switching (uptake then lock-in), compliance-amplified directionality
Active rectification under oscillatory forcing	Vibrated/acoustically driven/electromechanical platforms; droplets, films, and vibration-driven solid transport (stick–slip analogues)	Zero-mean periodic input + spatial anisotropy leads to net drift per cycle via asymmetric depinning / Young-force imbalance / direction-dependent dissipation	Forcing amplitude/acceleration threshold, frequency and waveform, texture scale relative to droplet, track/junction design, enclosure to reduce evaporation	Programmable droplet conveyors (routing, gating, synchronization), reconfigurable lab-on-surface operations, parallel solid manipulation via stick–slip

## Data Availability

No new data is generated.
